# Enhanced soil fertility, plant growth promotion and microbial enzymatic activities of vermicomposted fly ash

**DOI:** 10.1038/s41598-019-46821-5

**Published:** 2019-07-18

**Authors:** Zeba Usmani, Vipin Kumar, Pratishtha Gupta, Gauri Gupta, Rupa Rani, Avantika Chandra

**Affiliations:** Laboratory of Applied Microbiology, Department of Environmental Science and Engineering, Center of Mining Environment, Indian Institute of Technology (Indian School of Mines) Dhanbad, Dhanbad, Jharkhand India

**Keywords:** Environmental impact, Ecology

## Abstract

It is reported that coal consumption in the Asia-Pacific region is going to increase to about 87.2 percent by 2035. Management of coal combustion residues (CCRs) generated by industries is a major bottleneck towards handling the repercussions of coal usage. The present study investigates a management technique for these potentially hazardous wastes by means of vermicomposting. In the present investigation, studies were made on the effects of various concentrations of vermicomposted fly ash (VCF) added to agricultural soil, on the growth and yield of tomato (*Lycopersicon esculentum* Mill.) and brinjal (*Solanum melongena* L.) plants. The toxicity of trace elements in VCF were estimated using coefficient of pollution and potential ecological risk index, which revealed no apparent risks to the environment. A gradual increase in VCF concentrations in the agricultural soil improved the physico-chemical properties, enzymatic activities, microbial biomass, carbon and microbial population upto 90 days after sowing of seeds. The VCF amendments significantly (*p* < *0*.*05*) improved the soil quality (2.86% nitrogen and 1.05% Phosphorous) and germination percentage (82.22%) of seeds in *L*. *esculentum* and also in *S*. *melongena*. The results of this study reveal that, CCRs can be effectively managed in agriculture specially in developing economies.

## Introduction

The demand for electricity is increasing throughout the world and the trend is expected to continue in the years to come. About 70% of the electricity in India is generated through coal based thermal power plants, which produce approximately 65 million tons of fly ash (FA) in a year as a by-product^1^. The production of FA majorly depends on the coal quality, which comprises a fairly high proportion of ash that leads to 10–30% of FA formation^2^. In recent times, disposal of FA has become a chief concern globally. Moreover, this problem has become a serious apprehension in the developing countries and is generally carried out in landfills nearby the thermal power plants.

Utilization of FA in revegetating the landfill regions is an alternative for FA management, which serves both for stabilization and delivering an amiable landscape^3–6^. Additionally, this management technique possibly convalesces the physico-chemical properties of soil like pH, texture and water holding capacity (WHC). Supplement of alkaline FA, which has a pH above 9.0^7^, can decrease soil acidity to a level suitable for agriculture^8^, and can increase the accessibility of trace metals, SO_2_ and other nutrients^9^. However, direct application of FA to agricultural ground would not be quite advantageous to crops, due to little availability of most of the essential nutrient elements viz. nitrogen (N) and phosphorous (P), and a lower rate of FA degradation after its application in soil. Moreover, FA has a prevalence of heavy metals in the material and soluble forms^10^. FA comprises a high concentration of toxic heavy metals like Cr, Pb, Cd, Ni, Cu, Zn, etc.^11–13^.

Utilization of FA through vermicomposting is a crucial step towards environmental sustainability and retaining soil quality to reduce the dependency on agrochemical fertilizers. It is also an effective method for extenuation of metals from FA^10^. Earthworm species exhibiting vermicomposting (*Eisenia fetida*, *Eudrilus eugeniae* and *Lumbricus rubellus*) have an ability to increase the availability of key nutrient elements like phosphorous and nitrogen in FA, whilst reducing the solubility of heavy metals. Application of vermicomposted fly ash (VCF) to enhance crop productivity would not only be a resolution to the problem of FA disposal, but might also decline the use of chemical non-nitrogen fertilizers^14,15^.

There are almost no studies performed on the incorporation of VCF to the agricultural soil to determine the growth and yield of vegetable crops. In view of the above and to attain an efficient utilization of FA in agriculture, the present study deals with two main objectives: (i) to determine the variations in the physico-chemical properties of VCF amended soil at different rates of FA incorporation and (ii) to assess the growth and yield of two vegetable plants (*Lycopersicon esculentum* Mill. and *Solanum melongena* L.). The study also includes the quantification of photosynthetic pigments, shoot nitrogen and boron from both the plants. Photosynthesis and respiration rates were also estimated during the growth period of the plants.

## Results

### Physico-chemical properties of treated soil

The physico-chemical characteristics of treatments, before and at harvesting of *L*. *esculentum* are presented in Table 1. The bulk density of the treatments at the time of sowing was found to be lower as compared to the time of harvesting. The maximum cation exchange capacity (CEC) at the time of harvesting was observed for T6 i.e. 5.14 meq/100 g and minimal value of CEC was observed for T1 (4.67 meq/100 g). CEC values, total N and available P showed an increasing trend from the time of sowing to the time of harvest. The concentration of Mg was found to be higher at the time of harvesting compared to sowing. Mn concentration was maximum for T6 (25.35 mg/kg) and the concentration at the time of harvesting was higher compared to that of sowing for all the treatments (Table 2).

**Table 1:** Physico-chemical properties of vermicomposted fly ash amended soil at the time of sowing and harvesting of *Lycopersicon esculentum* and *Solanum melongena*.

Parameters	Days	T1	T2	T3	T4	T5	T6
Bulk density (g/cm^3^)	0	1.61 ± 0.05b	1.46 ± 0.02b	1.37 ± 0.01c	1.23 ± 0.02a	1.06 ± 0.02d	0.97 ± 0.01e
	120 (Le)	1.30 ± 0.01a	1.31 ± 0.01a	1.26 ± 0.02b	0.93 ± 0.07d	0.98 ± 0.01c	0.92 ± 0.006c
	120 (Sm)	1.32 ± 0.05a	1.34 ± 0.02a	1.28 ± 0.04b	0.96 ± 0.002c	0.94 ± 0.01c	0.89 ± 0.02c
Porosity (%)	0	41.71 ± 1.65d	44.26 ± 1.21c	46.38 ± 0.20b	47.96 ± 0.50b	48.71 ± 0.36b	50.86 ± 0.71a
	120 (Le)	48.11 ± 0.52b	48.89 ± 0.14b	47.38 ± 0.35c	48.52 ± 0.04b	50.41 ± 0.08a	50.67 ± 0.91a
	120 (Sm)	46.24 ± 0.66b	47.66 ± 0.28a	46.54 ± 1.05b	46.28 ± 0.55b	45.27 ± 1.12c	44.89 ± 0.06d
WHC (%)	0	40.51 ± 0.22e	42.88 ± 0.58d	45.37 ± 0.23c	47.13 ± 1.32b	48.05 ± 0.67b	54.50 ± 0.84a
	120 (Le)	44.51 ± 0.87 f	45.25 ± 0.44e	46.86 ± 0.20d	48.32 ± 0.10c	49.04 ± 0.57b	55.50 ± 1.10a
	120 (Sm)	43.75 ± 0.02e	45.78 ± 0.55d	45.42 ± 0.87d	47.76 ± 0.02b	46.84 ± 0.06c	48.32 ± 1.72a
CEC (meq/100 g)	0	3.88 ± 0.01d	4.03 ± 0.02d	4.10 ± 0.02c	4.15 ± 0.02c	4.61 ± 0.01b	4.88 ± 0.01a
	120 (Le)	4.67 ± 0.09c	4.81 ± 0.03b	4.12 ± 0.03e	4.21 ± 0.04d	4.72 ± 0.06b	5.14 ± 0.03a
	120 (Sm)	3.75 ± 0.05b	3.81 ± 0.02a	3.12 ± 0.002d	3.25 ± 0.06c	3.72 ± 0.02b	3.14 ± 0.08d
pH	0	6.56 ± 0.02e	6.78 ± 0.02e	7.01 ± 0.10d	7.14 ± 0.02c	7.36 ± 0.02b	7.52 ± 0.02a
	120 (Le)	6.96 ± 0.02c	7.47 ± 0.07d	7.82 ± 0.02b	7.83 ± 0.02b	7.95 ± 0.03a	7.96 ± 0.02a
	120 (Sm)	6.86 ± 0.01b	6.78 ± 0.02b	6.95 ± 0.05b	7.04 ± 0.02a	7.06 ± 0.04a	7.09 ± 0.05a
OC (%)	0	1.35 ± 0.03d	1.54 ± 0.02c	1.78 ± 0.02b	1.94 ± 0.02b	2.05 ± 0.03a	2.09 ± 0.02a
	120 (Le)	1.49 ± 0.06 f	1.56 ± 0.02e	1.99 ± 0.06d	2.08 ± 0.02c	2.17 ± 0.02b	2.34 ± 0.03a
	120 (Sm)	1.55 ± 0.07c	1.57 ± 0.03c	1.98 ± 0.002b	2.12 ± 0.05b	2.34 ± 0.06a	2.47 ± 0.02a
EC (dS/m)	0	3.15 ± 0.06d	3.69 ± 0.03c	3.80 ± 0.05b	3.85 ± 0.01b	3.97 ± 0.02a	4.07 ± 0.02a
	120 (Le)	3.70 ± 0.15d	3.92 ± 0.04c	4.00 ± 0.02bc	4.03 ± 0.03b	4.09 ± 0.02b	4.80 ± 0.05a
	120 (Sm)	3.85 ± 0.05c	3.97 ± 0.04c	4.02 ± 0.02b	4.05 ± 0.06b	4.10 ± 0.60a	4.12 ± 0.57a
Total N (%)	0	1.26 ± 0.01e	1.38 ± 0.02d	1.44 ± 0.03d	2.04 ± 0.03c	2.17 ± 0.02b	2.29 ± 0.03a
	120 (Le)	1.26 ± 0.01 f	2.52 ± 0.23c	2.06 ± 0.02e	2.44 ± 0.09d	3.51 ± 0.06a	2.86 ± 0.09b
	120 (Sm)	1.46 ± 0.01d	2.66 ± 0.33c	2.65 ± 0.15c	2.54 ± 0.02b	2.58 ± 0.05b	2.72 ± 0.03a
Available P (%)	0	0.31 ± 0.02c	0.37 ± 0.02c	0.39 ± 0.02c	0.47 ± 0.03b	0.58 ± 0.01b	0.88 ± 0.01a
	120 (Le)	0.42 ± 0.02e	0.40 ± 0.02e	0.54 ± 0.02d	0.59 ± 0.03c	0.73 ± 0.06b	1.05 ± 0.03a
	120 (Sm)	0.54 ± 0.002e	0.57 ± 0.01d	0.64 ± 0.03c	0.66 ± 0.002c	0.68 ± 0.02ab	0.70 ± 0.05a
Exc. Potassium (%)	0	0.21 ± 0.05 f	0.24 ± 0.02e	0.27 ± 0.02d	0.29 ± 0.05c	0.32 ± 0.06b	0.36 ± 0.05a
	120 (Le)	0.25 ± 0.02d	0.34 ± 0.02c	0.25 ± 0.02d	0.28 ± 0.03d	0.37 ± 0.02b	0.51 ± 0.03a
	120 (Sm)	0.28 ± 0.003e	0.31 ± 0.01d	0.33 ± 0.002d	0.36 ± 0.002c	0.39 ± 0.06b	0.41 ± 0.001a
Sulphate (%)	0	2.96 ± 0.14e	3.15 ± 0.03d	3.67 ± 0.02c	3.79 ± 0.03b	4.12 ± 0.04a	4.18 ± 0.01a
	120 (Le)	3.08 ± 0.02e	3.49 ± 0.05d	3.93 ± 0.03b	3.87 ± 0.02c	4.64 ± 0.05a	4.40 ± 0.03a
	120 (Sm)	3.25 ± 0.05 f	3.46 ± 0.02e	3.58 ± 0.09d	3.64 ± 0.03c	3.75 ± 0.01b	3.81 ± 0.02a

Le: *Lycopersicon esculentum*; Sm: *Solanum melongena*.

Values are in Mean ± SD; (n = 3).

Different letters in the same row represent significant differences in the physico-chemical parameters of different treatments comprising *L. esculentum* and *S. melongena* at *p* < *0.05* according to Duncan’s Multiple Range Test (One-way ANOVA followed by Tukey’s test).

**Table 2:** Metal concentrations (mg/kg) of vermicomposted fly ash amended soil during sowing and harvesting of *Lycopersicon esculentum* and *Solanum melongena*.

Metals	Days	T1	T2	T3	T4	T5	T6
Cu	0	1.50 ± 0.13d	1.86 ± 0.04c	1.86 ± 0.02c	1.96 ± 0.02b	2.16 ± 0.03b	2.57 ± 0.01a
	120 (Le)	1.78 ± 0.04 f	1.95 ± 0.01e	2.09 ± 0.01d	2.15 ± 0.02c	2.57 ± 0.03b	3.17 ± 0.05a
	120 (Sm)	1.45 ± 0.07e	1.52 ± 0.04d	1.66 ± 0.04c	1.68 ± 0.04c	1.71 ± 0.02b	2.10 ± 0.05a
Zn	0	3.27 ± 0.07c	4.57 ± 0.09c	4.84 ± 0.02b	4.89 ± 0.03b	5.00 ± 0.03b	5.28 ± 0.03a
	120 (Le)	3.44 ± 0.07d	4.85 ± 0.03c	4.94 ± 0.02b	4.99 ± 0.04b	4.87 ± 0.01c	5.99 ± 0.08a
	120 (Sm)	2.88 ± 0.50d	2.97 ± 0.02c	3.04 ± 0.06c	3.15 ± 0.52b	3.26 ± 0.64b	3.34 ± 0.60a
Cd	0	0.30 ± 0.021d	0.34 ± 0.02d	0.58 ± 0.12c	0.54 ± 0.05c	0.66 ± 0.02b	0.77 ± 0.01a
	120 (Le)	0.53 ± 0.04d	0.45 ± 0.04e	0.58 ± 0.02d	0.69 ± 0.02c	0.76 ± 0.02b	0.91 ± 0.05a
	120 (Sm)	0.62 ± 0.004c	0.57 ± 0.002d	0.62 ± 0.001c	0.75 ± 0.02b	0.78 ± 0.02b	0.84 ± 0.01a
Pb	0	1.91 ± 0.05d	2.04 ± 0.01d	3.10 ± 0.02c	3.12 ± 0.03c	3.19 ± 0.01b	3.79 ± 0.06a
	120 (Le)	2.13 ± 0.04e	2.33 ± 0.04d	3.14 ± 0.02c	3.16 ± 0.01c	3.26 ± 0.02b	3.95 ± 0.03a
	120 (Sm)	1.97 ± 0.02e	2.10 ± 0.05d	2.13 ± 0.03d	2.24 ± 0.02c	2.45 ± 0.06b	2.52 ± 0.03a
Ni	0	0.75 ± 0.03e	0.87 ± 0.02d	1.05 ± 0.02c	1.08 ± 0.02c	1.16 ± 0.01b	1.21 ± 0.11a
	120 (Le)	1.05 ± 0.01d	0.93 ± 0.02e	1.15 ± 0.02c	1.12 ± 0.02c	1.32 ± 0.07b	1.44 ± 0.02a
	120 (Sm)	0.92 ± 0.02e	0.98 ± 0.01e	1.05 ± 0.002d	1.15 ± 0.07c	1.22 ± 0.30b	1.34 ± 0.04a
Fe	0	258.33 ± 0.58e	272 ± 3.60d	287.33 ± 2.08c	282.84 ± 9.04c	292.51 ± 1.99b	315.37 ± 3.14a
	120 (Le)	265.66 ± 12.01e	278.67 ± 16.20d	297.07 ± 1.86c	303.96 ± 7.78b	311.72 ± 6.44b	325.88 ± 1.46a
	120 (Sm)	267.45 ± 15.74e	275.42 ± 23.65d	282.58 ± 16.43c	284.66 ± 8.45c	295.33 ± 11.67 b	312.57 ± 10.56a
Cr	0	0.38 ± 0.04e	0.55 ± 0.01d	0.62 ± 0.06c	0.67 ± 0.02c	0.78 ± 0.02b	0.93 ± 0.03a
	120 (Le)	0.47 ± 0.02d	0.75 ± 0.03c	0.74 ± 0.02c	0.74 ± 0.02c	0.94 ± 0.04b	1.03 ± 0.02a
	120 (Sm)	0.52 ± 0.03e	0.68 ± 0.02d	0.75 ± 0.01c	0.78 ± 0.004c	0.85 ± 0.05b	0.94 ± 0.01a
Mn	0	13.74 ± 0.05d	17.55 ± 0.10c	17.87 ± 0.08c	20.17 ± 0.05b	21.08 ± 0.04b	24.68 ± 1.00a
	120 (Le)	16.46 ± 0.75d	18.78 ± 0.17c	18.51 ± 0.06c	21.08 ± 0.74b	28.25 ± 0.53a	25.35 ± 0.49a
	120 (Sm)	15.42 ± 0.66d	16.34 ± 0.58c	17.33 ± 0.47c	18.73 ± 0.44b	18.85 ± 0.36b	19.27 ± 0.05a
Ca*	0	0.10 ± 0.015e	0.24 ± 0.02d	0.3 ± 0.02c	0.34 ± 0.02c	0.45 ± 0.01b	0.67 ± 0.02a
	120 (Le)	0.14 ± 0.03d	0.47 ± 0.03c	0.47 ± 0.01c	0.45 ± 0.02c	0.58 ± 0.02b	0.83 ± 0.02a
	120 (Sm)	0.18 ± 0.02d	0.24 ± 0.02c	0.27 ± 0.05c	0.32 ± 0.04b	0.35 ± 0.02b	0.38 ± 0.02a
Mg*	0	0.083 ± 0.002e	0.09 ± 0.003d	0.15 ± 0.01c	0.18 ± 0.01c	0.27 ± 0.02b	0.41 ± 0.04a
	120 (Le)	0.10 ± 0.00e	0.11 ± 0.002e	0.18 ± 0.01d	0.27 ± 0.01c	0.36 ± 0.03b	0.43 ± 0.02a
	120 (Sm)	0.12 ± 0.004c	0.16 ± 0.005c	0.24 ± 0.03b	0.31 ± 0.002a	0.34 ± 0.001a	0.37 ± 0.01a

*Le*: *Lycopersicon esculentum*; *Sm*: *Solanum melongena*; Ca* and Mg*: Values of Ca and Mg are in percentage (%).

Values are in Mean ± SD; (n = 3).

Different letters in the same row represent significant differences in the mean of the metal concentrations of different treatments comprising *L*. *esculentum* and *S*. *melongena* at *p* < *0*.*05* according to Duncan’s Multiple Range Test (One-way ANOVA followed by Tukey’s test).

In the case of *S*. *melongena*, bulk density was higher in the treatments at the time of sowing compared to that of harvesting. The bulk density of the treatments ranged from 0.89–1.34 g/cm^3^ and maximum bulk density was observed for T2 (Table 1). The metal concentrations at 90 days after sowing (DAS) showed the following trend: T6 > T5 > T4 > T3 > T2 > T1 (Table 2) Concentrations of Ca and Mg were found to be higher at 90^th^ DAS compared to the 0^th^ day of sowing of seeds. The maximum concentration of Mg was observed for treatment T6 (0.37) while, the minimum concentration was observed for T1 (0.12) in the case of *S*. *melongena* (Table 2).

**Figure 1:**
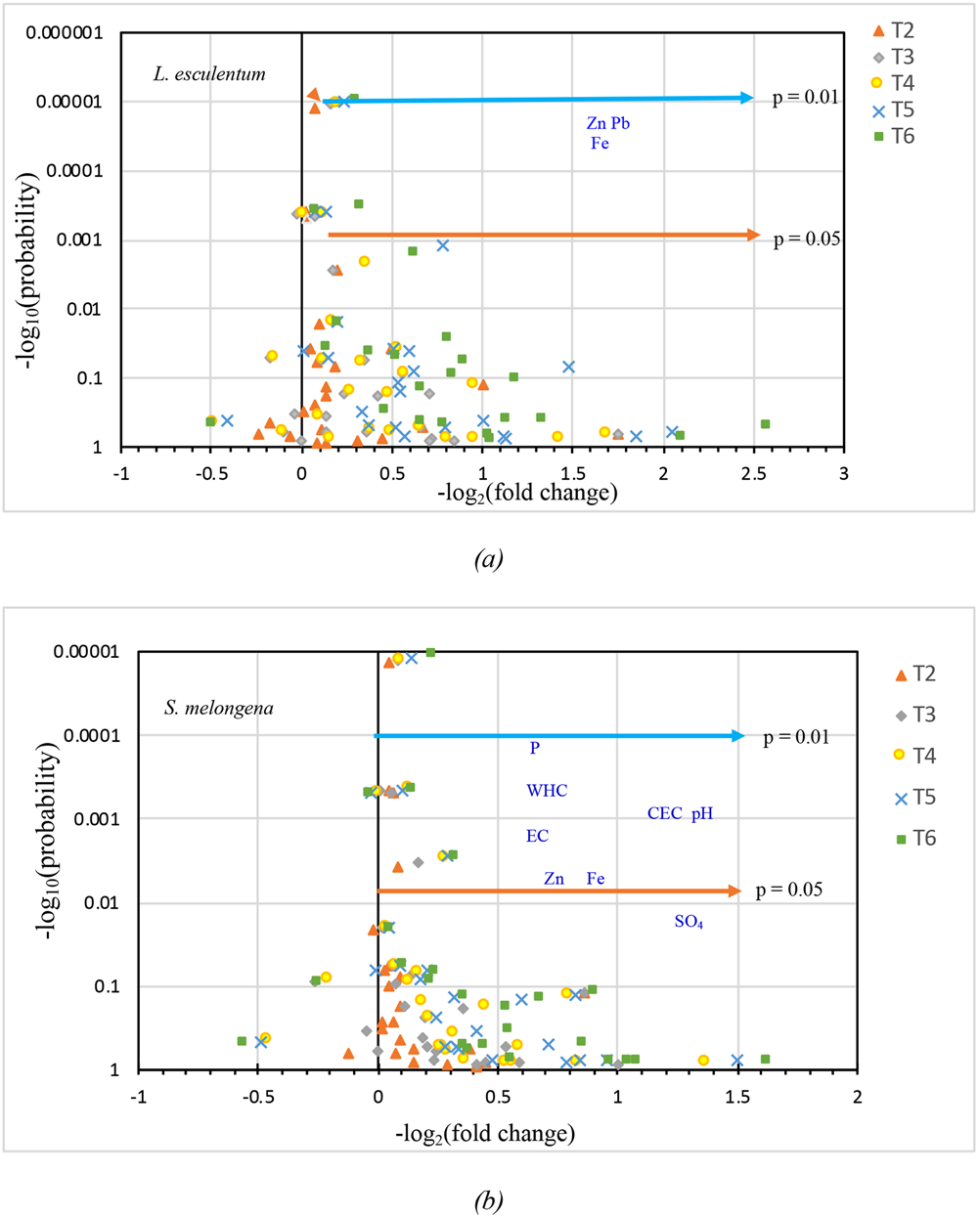
A volcano plot representing the expression of vermicomposted fly ash amended soil parameters under five different treatments scheme. Illustration: The effects of vermicomposted fly ash amended with soil before and after harvesting of *Lycopersicon esculentum* and *Solanum melongena* were enumerated by computing the average of treatment T1 (Control) and treatments: T2, T3, T4, T5, and T6. The X-axis represents logarithmic changes in soil physicochemical and nutritional attributes over basic soil, and the Y-axis represents the statistical significance of the difference based on one-way ANOVA considering the effects of a nutrient management scheme. Every individual attribute is displayed as a point. The compounds for which strong statistical differences (*p* < *0*.*01*) were found are presented with names. Av: Available; P: porosity; WHC: water holding capacity; EC: Electrical conductivity; CEC: Cation exchange capacity.

A volcano plot depicting the relationship between various physico-chemical parameters after harvesting of both the crops are depicted in Fig. 1(a,b). In *L*. *esculentum*, parameters such as Available P, WHC, Zn, Pb, Fe, Sulphate and Mn were observed to be very significant (*p* < 0.01) points of interest which displays both high-level statistical significance (−log 10 of p values, y-axis) and great magnitude fold changes (x-axis) (Fig. 1a). The parameters such as CEC, pH and EC were obtained with values having *p* < 0.05 (statistically significant).

In the case of *S*. *melongena*, parameters such as P, WHC, EC, CEC and pH were observed to have strong statistical differences (*p* < 0.01), while Zn and Fe were found to be moderately significant with values having *p* ≤ 0.05 (Fig. 1b).

### Dehydrogenase and Alkaline Phosphatase activities

**Figure 2:**
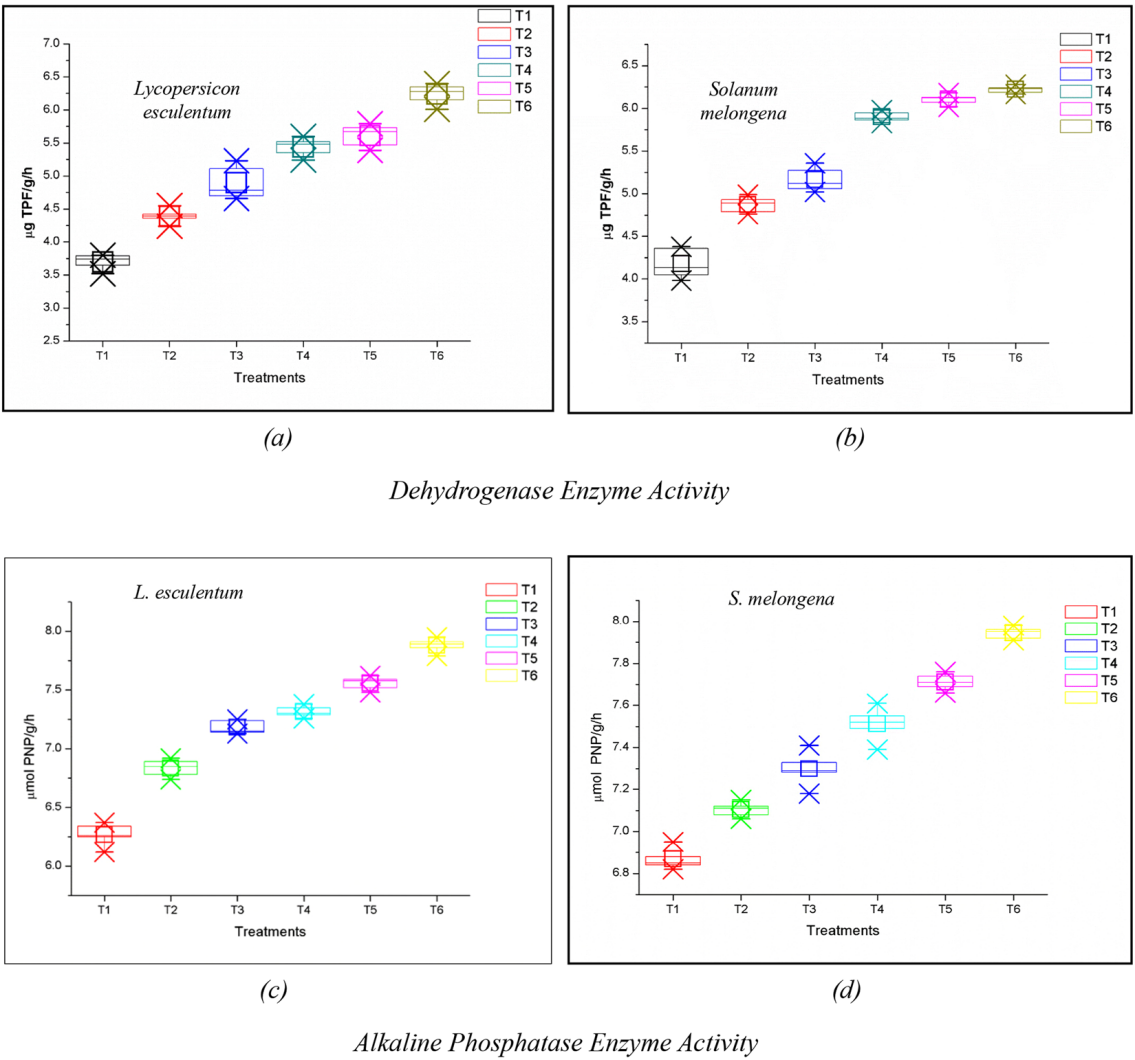
Variations in the dehydrogenase enzyme and alkaline phosphatase enzyme activity of treatments at the time of harvesting of *Lycopersicon esculentum* and *Solanum melongena*.

Variations in the dehydrogenase activity were observed during harvesting of *L*. *esculentum* and *S*. *melongena*. Dehydrogenase activity was higher in treatments comprising *L*. *esculentum* compared to *S*. *melongena*. The trend of the dehydrogenase action in the treatments was as follows: T6 > T5 > T4 > T3 > T2 > T1 both in the case of *L*. *esculentum* and *S*. *melongena*. Maximum dehydrogenase activity was observed for T6 in both *L*. *esculentum* (ranging from 6.01–6.4 µg TPF/g/h) and *S*. *melongena* (6.17–6.24 µg TPF/g/h) (Figs. 2a,b).

Alkaline phosphatase activity displayed an increase in trend with an increase in concentration of VCF in both the treatments comprising *L*. *esculentum* and *S*. *melongena* (Fig. 2c,d). The activity was found to be lower in treatments comprising soil alone. The phosphatase activity in *L*. *esculentum* was higher for treatments T5 and T6 while, the lower enzyme activity was observed for T1 and T2 (Fig. 2c). In *S*. *melongena*, maximum phosphatase activity was observed for T6 (7.89 µmol PNP/g/h) while minimum activity was observed for T1 (6.37 µmol PNP/g/h) (Fig. 2d).

### Variation in bacterial population among treatments

The trend for phosphate solubilizing bacteria (PSB) population was as follows: T6 > T5 > T4 > T3 > T2 > T1 at 90 DAS of *L*. *esculentum* (Table 3). In *S*. *melongena*, the maximum PSB population was observed for T6 (116 × 10^4^ cfu g^−1^) while, minimum was obtained for T1 (14 × 10^4^ cfu g^−1^). Significant differences (*p* < *0*.*05*) were detected in populations of *Azotobacter* among the several treatments. The maximum *Azotobacte*r population was observed for T6 (104 × 10^4^ cfu g^−1^) in the case of *L*. *esculentum*.

**Table 3:** Bacterial Population in soil amended with vermicomposted fly ash before (0^th^ day) and after (120^th^ day) harvesting of *Lycopersicon esculentum* and *Solanum melongena*.

Days/Crop	Unit	T1	T2	T3	T4	T5	T6
*Phosphate solubilizing bacteria*							
0^th^ day (*Le*/*Sm*)	cfu = ---×10^4^	03 ± 0.002 Cc	04 ± 0.001Cb	04 ± 0.002Cb	04 ± 0.05Cb	05 ± 0.003Ca	05 ± 0.006Ca
90^th^ day (*Le*)		16 ± 0.05Af	78 ± 0.54Ae	88 ± 0.25Ad	102 ± 1.25Ac	112 ± 0.58Ab	123 ± 1.54Aa
90^th^ day (*Sm*)		14 ± 0.02Bf	74 ± 0.36Be	85 ± 0.57Bd	97 ± 0.75Bc	106 ± 1.12Bb	116 ± 0.82Ba
*Azotobacter*							
0^th^ day (*Le*/*Sm*)	cfu = ---×10^3^	07 ± 0.001 Cc	08 ± 0.005Cb	08 ± 0.03Cb	08 ± 0.002Cb	09 ± 0.05Ca	09 ± 0.02Ca
90^th^ day (*Le*)		12 ± 0.20Af	69 ± 0.38Ae	75 ± 0.55Ad	86 ± 0.45Ac	95 ± 0.27Ab	104 ± 1.02Aa
90^th^ day (*Sm*)		10 ± 0.08Bf	65 ± 0.54Be	73 ± 0.65Bd	84 ± 0.52Bc	92 ± 0.25Bb	101 ± 1.46Ba
*Potash mobilizing bacteria*							
0^th^ day (*Le*/*Sm*)	cfu = ---×10^2^	04 ± 0.001 Cd	06 ± 0.003 Cc	08 ± 0.04Cb	08 ± 0.002Cb	9 ± 0.04Ca	9 ± 0.05Ca
90^th^ day (*Le*)		09 ± 0.25Af	18 ± 0.42Ae	21 ± 0.50Ad	24 ± 0.52Ac	28 ± 0.09Ab	38 ± 0.45Aa
90^th^ day (*Sm*)		07 ± 0.03Be	15 ± 0.55Bd	19 ± 0.68Bc	21 ± 0.87Bc	23 ± 0.51Bb	31 ± 0.17Ba

Le: *Lycopersicon esculentum*; Sm: *Solanum melongena*; cfu: colony forming unit.

Values are in Mean ± SD; (n = 3).

Different small letters along the row represents significant differences in the bacterial count of different treatments of Le and Sm at a particular time interval (sowing and harvesting) according to Duncan’s Multiple Range Test (One-way ANOVA) at *p* < *0.05*.

Different capital letters along the column represents significant differences in the count of phosphate solubilizing bacteria, *Azotobacter* and Potash mobilizing bacteria in soil of *Le* and *Sm* before and after plantation within the same treatment, respectively, according to Duncan’s Multiple Range Test (ANOVA) at *p* < *0.05*.

The population of potash mobilizing bacteria was observed to be significantly (*p* < *0*.*05*) lesser than PSB and *Azotobacter* at the time of harvesting of *L*. *esculentum* and *S*. *melongena*. The population of potash mobilizing bacteria was observed to be lower for T1 at the time of sowing and harvesting of *L*. *esculentum* and *S*. *melongena* at 90 days after sowing of *L*. *esculentum* (Table 3). For Treatment T6, the trend for Potash mobilizing bacteria was higher at the time of harvesting of *L*. *esculentum* compared to *S*. *melongena* at the 0^th^ day of sowing.

### Evaluation of PGP traits

All bacterial strains tested were positive to produce indole acetic acid (IAA) (Table 4). In *L*. *esculentum*, maximum siderophores production was observed for PSB (28.57), followed by *Azotobacter* (12.67) and potash mobilizing bacteria (3.55) (Table 4). In the case of *S*. *melongena*, the isolated strains of PSB, showed maximum siderophores production (25.45). All the isolated bacterial strains tested positive to produce ammonia. The details on PGP characteristics of the isolates are listed in Table 4.

**Table 4:** Plant Growth Promoting (PGP) properties of selected isolates during harvesting *of L*. *esculentum* and *S*. *melongena* for treatment comprising 15% vermicomposted fly ash (T6).

Bacteria	Number of strains (%)	IAA Production (%)	Siderophores (%)	HCN test (%)	Ammonia production (%)	P-Solubilization (%)
*Lycopersicon esculentum*						
PSB	23	100	28.57	0	57.14	100
*Azotobacter*	18	100	12.67	0	47.28	15
Potash mobilizing	09	100	3.55	0	63.65	2
*Solanum melongena*						
PSB	21	100	25.45	0	52.65	98
*Azotobacter*	15	100	10.28	0	40.53	12
Potash mobilizing	06	100	2.36	0	58.20	2

PSB: Phosphate solubilisation bacteria; IAA: Indole acetic acid; HCN: Hydrogen cyanide.

Furthermore, PSB showed almost 100% phosphate solubilization during harvesting of *L*. *esculentum* and *S*. *melongena* while, *Azotobacter* and potash mobilizing bacteria showed lower rates of phosphorous solubilization (Table 4). Thus, the bacteria present in treatment T6 after harvesting of *L*. *esculentum* and *S*. *melongena* displayed a wide variety of activities which are essential for plant growth such as production of IAA, solubilisation of phosphates and production of ammonia and siderophores.

### Microbial biomass carbon

**Figure 3:**
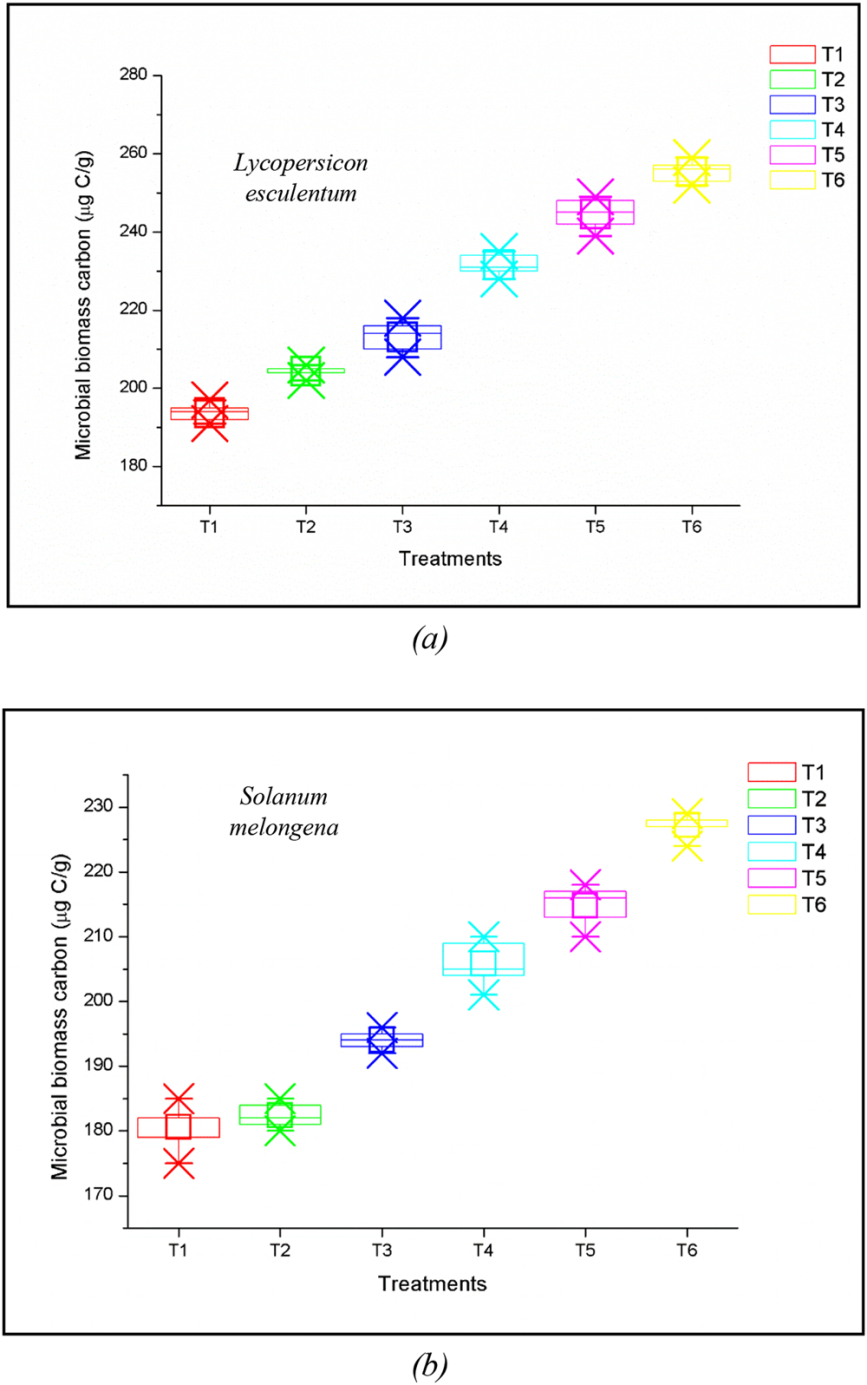
Variations in the microbial biomass carbon of treatments at the time of harvesting of *Lycopersicon esculentum* and *Solanum melongena*.

Microbial biomass carbon (MBC) showed a direct relation with the concentration of VCF. Higher MBC values were observed for T5 and T6 in both *L*. *esculentum* and *S*. *melongena* (Fig. 3a,b). MBC values were found to be lower for T1. The trend for MBC among the treatments was T6 > T5 > T4 > T3 > T2 > T1.

### Effects of application of vermicomposted fly ash on the plant growth

#### Seed germination

*L*. *esculentum* plants showed positive response towards VCF soil amendment thus, exhibiting luxuriant growth. No visual symptoms related to toxicity of the FA, or to deficit of a particular nutrient had effects on the rate of seed germination. The results showed that the rate of seed germination significantly (*p* < 0.05) increased with the increase in rates of application of VCF (Table 5). The maximum increase in seed germination was found for the treatment, T6 (i.e. 8.56). The rate of seed germination of *S*. *melongena* was found to significantly increase (*p* < 0.05) with the increase in concentration of VCF showing the following trend T6 (5.56) > T5 (4.02) > T4 (3.48) > T3 (2.13) > T2 (1.89) > T1 (1.75) (Table 5).

**Table 5:** Effects of vermicomposted fly ash on the rate of seed germination, number of flowers, fruits per pot, weight of fruits, yield of fruits per plant and percent increase of yield over control in *L*. *esculentum* and *S*. *melongena*.

Treatments	Increase in Seed Germination over Control (%)	Rate of Seed Germination	Number of Flowers	No. of fruits per pot	Weight of fruits (g)	Yield of fruit per plant (g/plant)
*Lycopersicon esculentum*						
T1	—	6.75 ± 0.05f	3.00 ± 0.58e	5.00 ± 1.00e	161.98 ± 36.42f	126.14 ± 5.99f
T2	37.78	6.89 ± 0.42e	7.00 ± 1.00d	11.00 ± 1.00d	260.47 ± 44.52e	326.17 ± 22.50e
T3	53.30	7.13 ± 0.66d	13.00 ± 2.08c	14.00 ± 3.00c	323.82 ± 12.11d	668.54 ± 16.31d
T4	60.00	7.48 ± 0.54c	14.00 ± 2b	20.00 ± 4.04b	632.84 ± 53.86c	859.00 ± 5.99c
T5	73.33	8.02 ± 0.18b	15.00 ± 3.51b	21.00 ± 2.65b	885.59 ± 66.89b	922.48 ± 13.14b
T6	82.22	8.56 ± 0.53a	25.00 ± 2.52a	23.00 ± 3.06a	1334.91 ± 9.26a	952.29 ± 10.60a
*Solanum melongena*						
T1	—	1.75 ± 0.05f	1.00 ± 0.58 f	7.00 ± 1.00d	724.21 ± 35.23f	618.35 ± 42.84f
T2	16.92	1.89 ± 0.42e	2.00 ± 1.00e	7.00 ± 0.53d	827.29 ± 16.83e	836.12 ± 44.42e
T3	21.54	2.13 ± 0.66d	4.00 ± 1.53d	8.00 ± 1.00c	1266.39 ± 16.67d	1078.56 ± 8.08d
T4	26.15	3.48 ± 0.54c	5.67 ± 1.53c	8.00 ± 0.58b	1646.68 ± 10.35c	1142.59 ± 43.39c
T5	29.2	4.02 ± 0.18b	6.00 ± 2.00b	9.00 ± 1.53b	1800.61 ± 45.38b	1249.07 ± 48.55b
T6	32.30	5.56 ± 0.53a	8.00 ± 1.00a	11.00 ± 0.58a	1984. 81 ± 61.48a	1293.13 ± 43.97a
*Results of two-way ANOVA test*						
*A*	—	—	4.72*	10.47**	7.58**	6.43*
*F*	—	—	32.29***	23.51**	14.25***	23.57**
*A x F*	—	—	3.15*	0.92^NS^	0.48^NS^	0.74^NS^

Values are in Mean ± SD, n = 3

Levels of significance: ***p < *0*.*001*; **p < *0*.*01*; *p < *0*.*05*; NS = not significant (Two-way ANOVA); (−): Two-way ANOVA not applied.

Different letters in the same column denote significant differences (*p* < *0*.*05*) in the rate of seed germination, number of flowers, number of fruits per pot, weight of fruits, yield of fruits per plant of *L*. *esculentum and S*. *melongena*, respectively in different treatments (One-way ANOVA; Tukey’s test).

#### Effects on shoot and root length and weight and number of leaves

**Figure 4:**
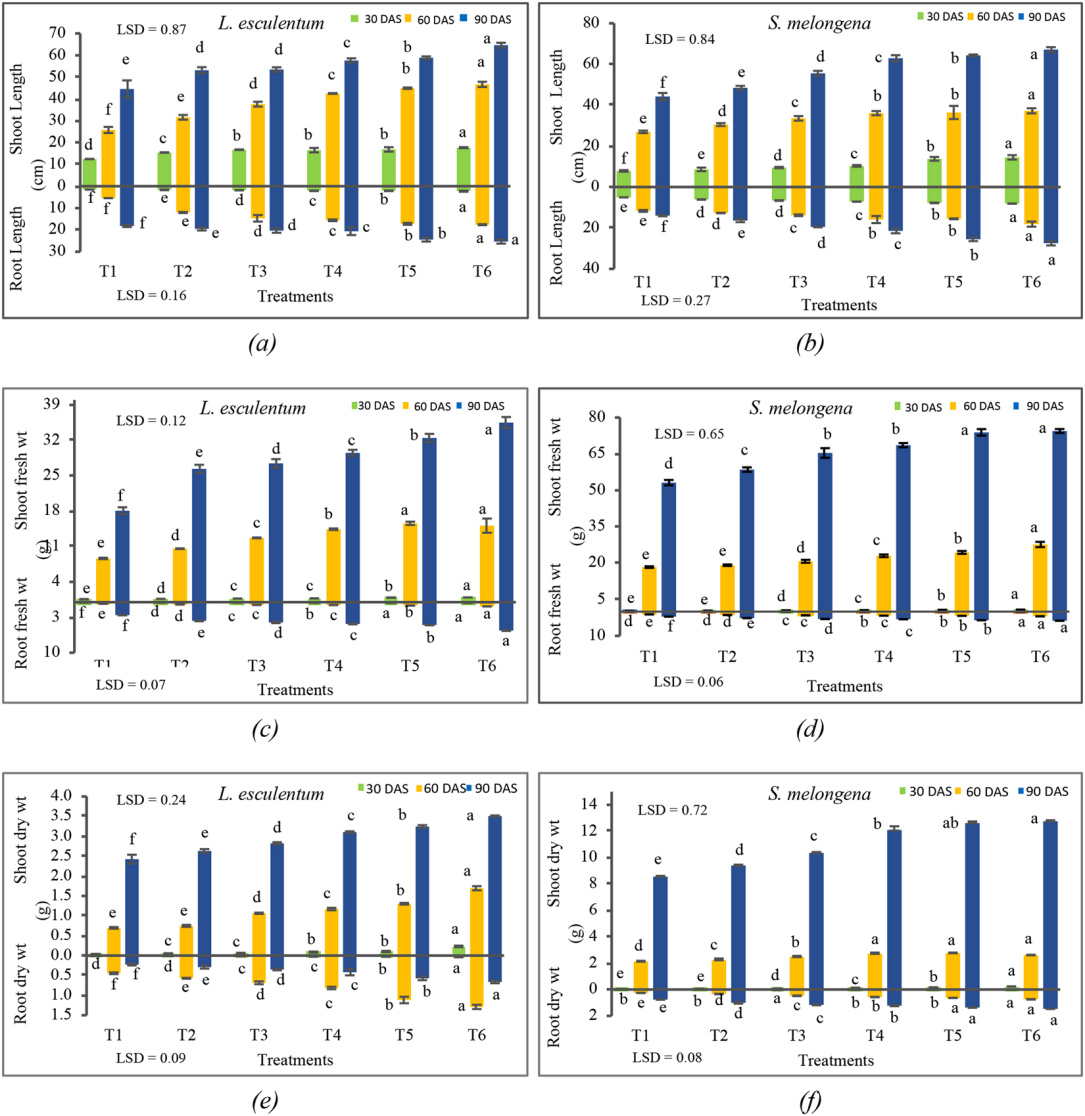
Effects of vermicomposted fly ash on the shoot and root length, shoot and root fresh weight and shoot and root dry weight of *Lycopersicon esculentum* and *Solanum melongena* at different .durations after sowing in different treatments Illustration: Values are in Mean ± SD; (n = 3). Means with bars of different colours followed by the same letter are not significantly different at *p* < *0*.*05* (One-way ANOVA followed by Least significance difference test). The normality and homogeneity of variances were verified, respectively, by Shapiro-Wilk and Levene values at *p* > *0*.*05*.

The data on shoot and root length of *L*. *esculentum* at different growth stages as influenced by bio-formulations are presented in Fig. 4a. The shoot length displayed an upsurge in trend as per the duration of sowing of seeds. At the time of harvesting (90 DAS), the shoot length depicted an increase with an increase in the rates of VCF amendment to the agricultural soil. An increase in the trend of root length was also observed with increase in the rates of VCF (Fig. 4b).

The increase in the weight of shoots with increased rates of application of VCF and duration of sowing was observed (Fig. 4c–f)). The dry and fresh weight of root and shoot of *L*. *esculentum* were found to increase with the duration of sowing. The maximum increase in shoot fresh weight was observed for T6 (35.50 g) at 90 days after sowing and maximum increase in shoot dry weight was also observed for T6 (3.51 g). The root fresh and dry weight of *L*. *esculentum* were also observed to be maximized for treatment T6 at 90 DAS with values 5.61 g and 0.66 g respectively (Fig. 4c,e).

**Figure 5:**
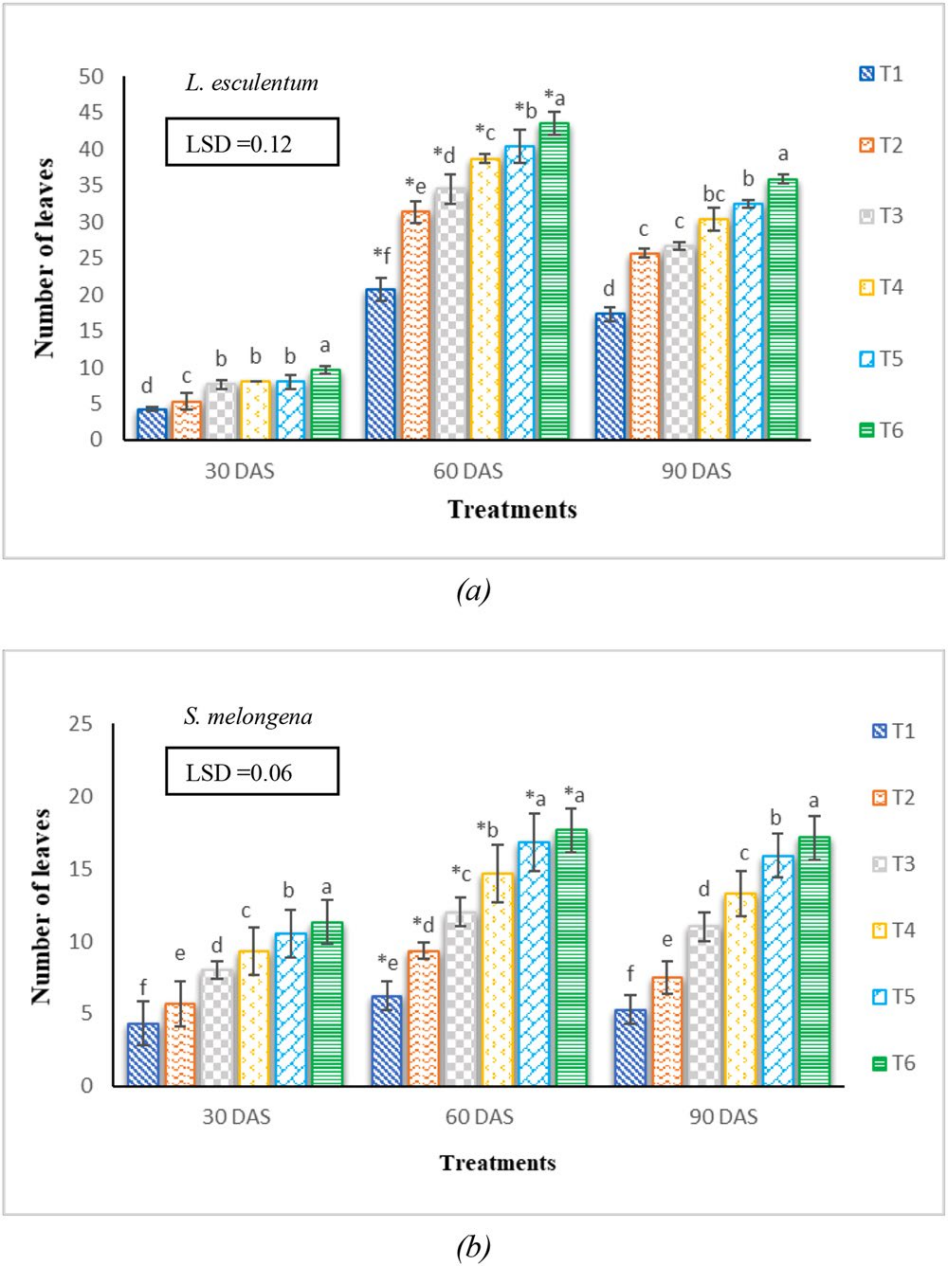
Effects of vermicomposted fly ash applied at different rates on the number of leaves in *Lycopersicon esculentum* and *Solanum melongena* at different durations. Illustration: Values are in Mean ± SD; (n = 3). Means with bars of different patterns followed by the same letter are not significantly different at *p* < *0*.*05* (One-way ANOVA followed by LSD test). The normality and homogeneity of variances were verified, respectively, by Shapiro-Wilk and Levene values at *p* > *0*.*05*. *Repeated measures ANOVA was applied to determine significant differences between the number of leaves in different treatments at different durations.

The number of leaves increased with the increase in rates of application of VCF to the treatments (Fig. 5a,b). A maximum number of leaves were observed for the treatments comprising 15% VCF added to agricultural soil, while, minimum in treatments comprising 3% VCF.

#### Effects on the number of flowers and fruits in *Lycopersicon esculentum* and *Solanum melongena*

Significant differences were observed in the flower count among various treatments (Table 5) and maximum number of flowers were found in case of treatment, T6. The number of fruits per pot was maximum for T6 (23) while, minimum for T1 (5). The weight of the fruits showed an increase with an increase in the rates of application of VCF proving FA to have good fertilizing activity. Significant (*p* < *0*.*05*) variances were found in the yield of fruit per plant along the treatments. The yield of fruits per *L. esculentum* plant was high for treatments T5 (922.48 g/plant) and T6 (952.29 g/plant).

**Figure 6:**
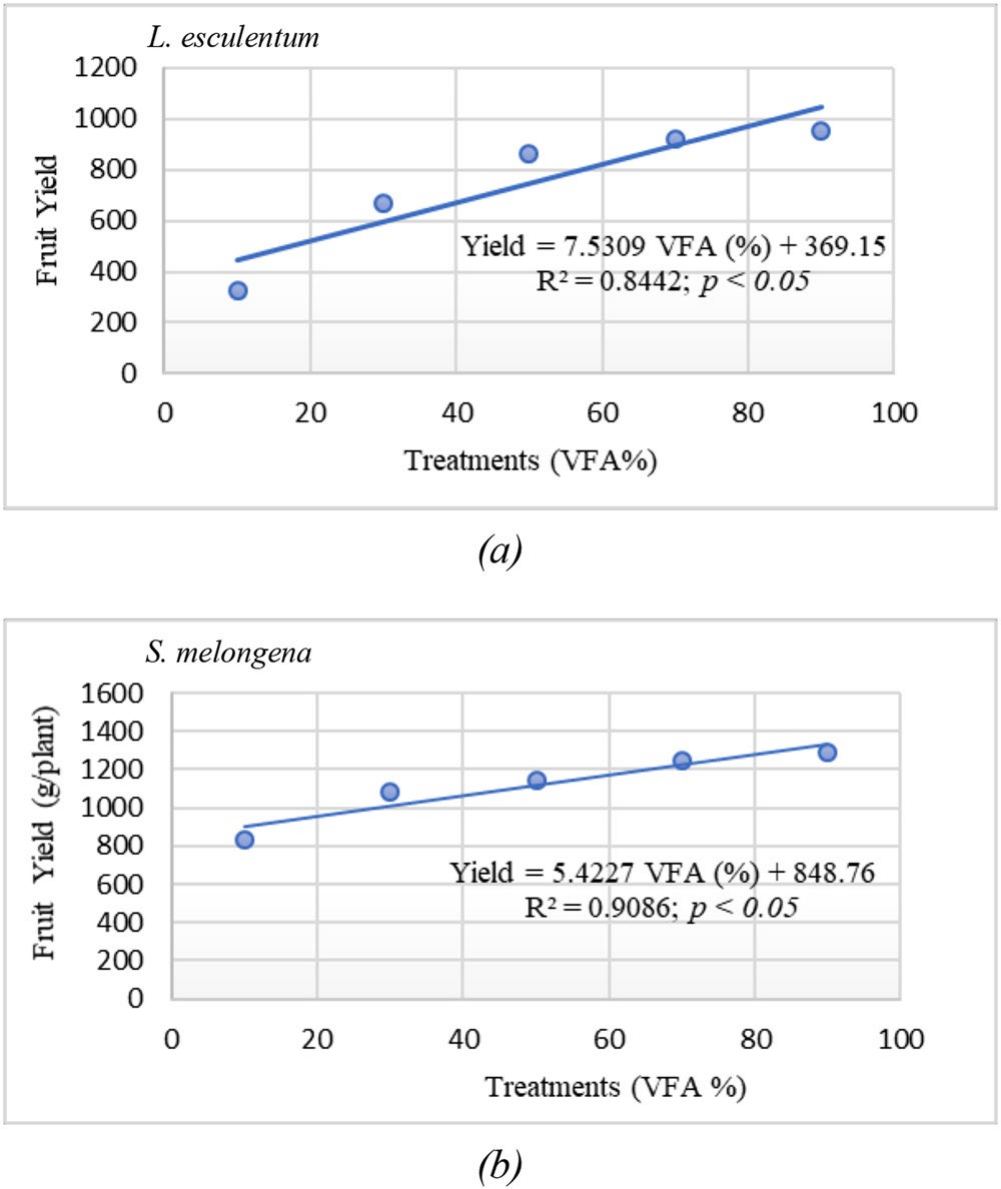
The relationship between vermicomposted fly ash concentrations incorporated in soil and yield of *Lycopersicon esculentum* and *Solanum melongena* respectively.

The number of *S*. *melongena* fruits per pot was found to be maximum in T6 and minimum in T1 (Table 5). Maximum yield in fruits per plant was observed for T6 (1293.13 g/plant) followed by T5 (1249 g/plant) and T4 (1293.13 g/plant). Regression equations depicting the relationship between VCF concentration and fruit yield were derived for both the crops and are depicted in Fig. 6(a,b).

### Effects on photosynthetic pigments, boron, shoot nitrogen and total phenols

In *L*. *esculentum*, the maximum concentration of chlorophyll a (749.37 µg/g) and chlorophyll b (462.55 µg/g) were found for T6 (Table 6). The concentration of carotenoids showed the following trend: T6 > T5 > T4 > T3 > T2 > T1. Carotenoid concentration was found to be minimum for T1 (5.85 µg/g) and maximum for T6 (7.83 µg/g). The VCF on application to the agricultural soil at the rate of 15% by weight showed a maximum concentration of carotenoids, thus verifying it to have good fertilizing ability. The concentration of boron in the treatments comprising VCF as the amendment was found to be maximum for treatment T6 (447.98 µg/g). Shoot nitrogen was found to vary significantly (*p* < *0*.*05*) along the treatments. Total phenols and boron showed a direct relationship with the increase in the concentration of VCF.

**Table 6:** Effects of vermicomposted fly ash amendment on photosynthetic pigments, boron, shoot nitrogen and total phenol in leaves of *Lycopersicon esculentum and Solanum melongena* plant during harvesting.

Treatment	Chlorophyll a (µg/g)	Chlorophyll b (µg/g)	Carotenoids (µg/g)	Boron (µg/g)	Shoot Nitrogen (µg/g)	Total Phenols (mg/100 g dry weight)
*Lycopersicon esculentum*						
T1	655.22 ± 2.56f	321.71 ± 3.67f	5.85 ± 0.19f	204.14 ± 1.72f	1.55 ± 0.03a	325.95 ± 7.24f
T2	664.28 ± 1.37e	360.73 ± 7.44e	6.22 ± 0.03e	222.17 ± 3.97e	1.43 ± 0.03b	408.82 ± 3.65e
T3	683.42 ± 4.11d	374.60 ± 6.93d	6.94 ± 0.04d	274.57 ± 3.50d	1.32 ± 0.04c	423.94 ± 4.01d
T4	704.16 ± 9.30c	397.44 ± 0.95c	7.27 ± 0.08c	312.32 ± 7.54c	1.20 ± 0.04d	434.11 ± 7.25c
T5	729.94 ± 4.45b	404.33 ± 7.69b	7.53 ± 0.08b	412.35 ± 6.09b	1.05 ± 0.03e	457.11 ± 1.08b
T6	749.37 ± 6.32a	462.55 ± 17.57a	7.83 ± 0.04a	447.98 ± 7.66a	0.87 ± 0.04f	513.98 ± 1.23a
*Solanum melongena*						
T1	691.21 ± 2.41e	679.48 ± 18.75e	6.36 ± 0.26d	246.44 ± 11.15e	2.76 ± 0.10a	336.46 ± 7.04f
T2	715.21 ± 18.3d	691.99 ± 57.83d	6.60 ± 0.64d	276.25 ± 8.89d	2.46 ± 0.11b	415.53 ± 5.04e
T3	744.50 ± 30.28c	726.02 ± 87.18c	7.38 ± 0.22c	304.63 ± 7.30c	2.15 ± 0.03d	439.29 ± 11.71d
T4	757.26 ± 51.96c	756.74 ± 32.29b	7.63 ± 0.45c	330.66 ± 10.51b	2.22 ± 0.32c	469.36 ± 10.80c
T5	791.95 ± 30.44b	763.34 ± 85.17b	8.45 ± 0.28b	332.14 ± 11.90b	1.80 ± 0.14e	481.04 ± 10.80b
T6	811.59 ± 14.02a	822.84 ± 18.25a	9.24 ± 0.36a	376.37 ± 7.48a	1.67 ± 0.015f	497.61 ± 77.11a
*Results of two-way ANOVA test*						
*A*	225***	247.82***	138.65**	210.57***	176.44**	95.88**
*F*	135.11**	87.56**	75.42**	63.08**	82.59**	48.36**
*A x F*	12.08**	5.82**	7.63**	1.95^NS^	3.77**	7.63**

DAS: Days after sowing.

Values are in Mean ± SD, n = 3.

Levels of significance: ***p < *0*.*001*; **p < *0*.*01*; *p < *0*.*05*; NS = not significant (Two-way Anova).

Different letters in the same column denote significant differences (*p* < *0*.*05*) in the concentrations of chlorophyll a, chlorophyll b, carotenoids, boron, shoot nitrogen and total phenol in *Lycopersicon esculentum* and *Solanum melongena* plants respectively (One-way ANOVA; Tukey’s test).

In *S*. *melongena*, the concentration of chlorophyll a was observed to increase significantly (*p* < *0*.*05*) with an increase in the concentration of VCF. The maximum concentration of carotenoid was observed in the treatment T6 (376.37 µg/g) while, minimum concentration in T1 (822.84 µg/g) (Table 6). The trend in boron concentration had direct relations with the application rates of VCF of agricultural soil. Total phenols also showed a significant (*p* < *0*.*05*) increase in trend with concentrations varying among the treatments as T1 (336.46 mg/100 g), T2 (415.53 mg/100 g), T3 (439.29 mg/100 g), T4 (469.29 mg/100 g), T5 (481.04 mg/100 g), T6 (447.61 mg/ 100 g).

### Effects on photosynthesis and respiration rates

**Figure 7:**
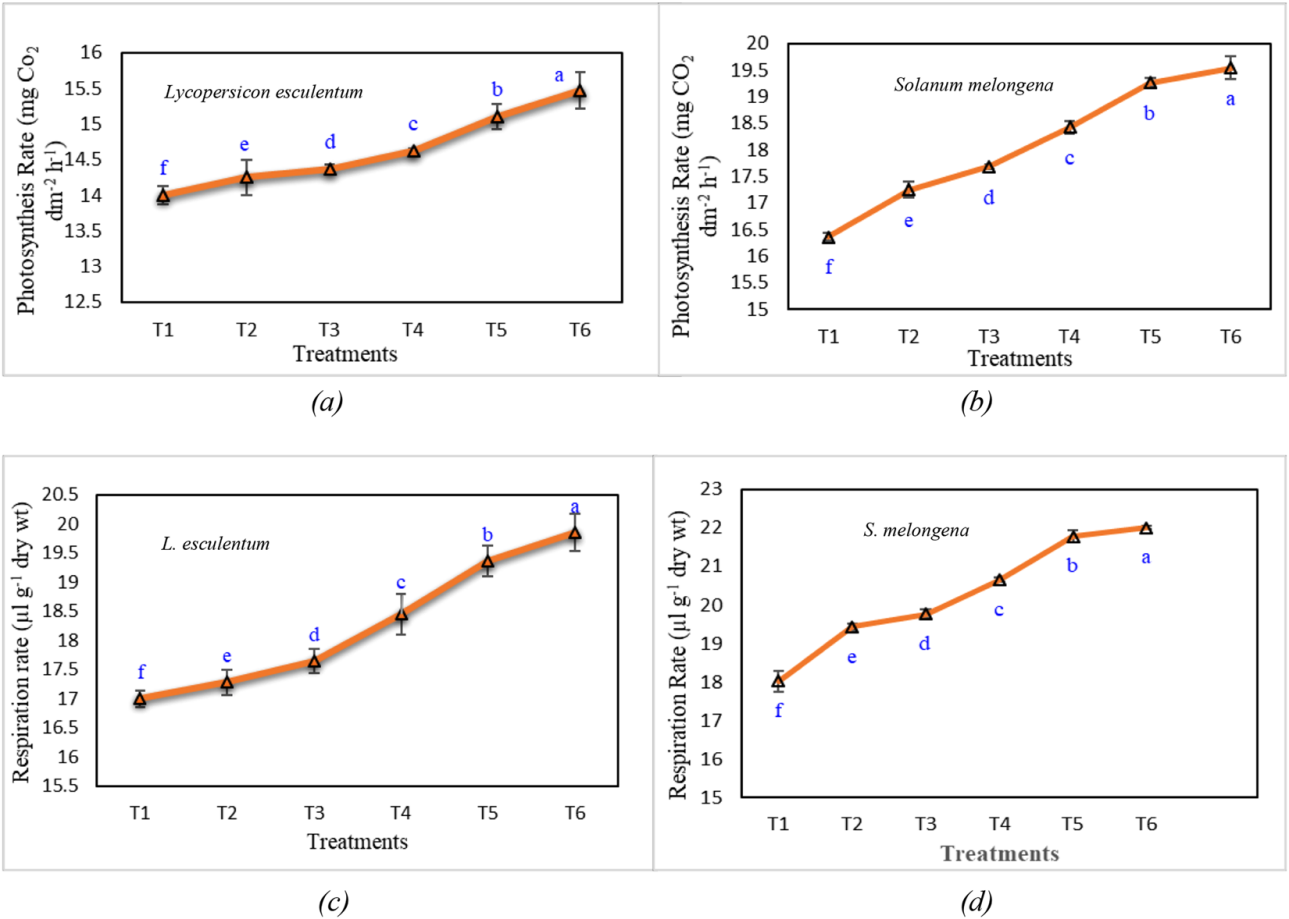
Photosynthesis and Respiration rate during harvesting of *Lycopersicon esculentum* and *Solanum melongena* dusted at different rates of vermicomposted fly ash. Illustration: Values are in Mean ± SD; (n = 3). Different letters at each data point of the line graph depict significant differences in the photosynthetic and respiration rates respectively at *p* < *0*.*05* (One-Way ANOVA). The normality and homogeneity of variances were verified, respectively, by Shapiro-Wilk and Levene values at *p* > *0*.*05*.

VCF deposition augmented the apparent rate of photosynthesis in both the crops, approaching a maximum of 15.5 mg CO_2_ dm^−2^ h^−1^ in *L*. *esculentum* (Fig. 7a) and 19.5 mg CO_2_ dm^−2^ h^−1^ in *S*. *melongena* (Fig. 7b) at 90 DAS. The increase in the rate of photosynthesis was attributed to increased foliar temperatures, that might have hastened photosynthetic activity. In both crops, rate of respiration increased with upsurge in concentration of VCF amended soil. Due to the maximum increase in plant growth and several greener leaves in T6, respiration rates in leaves of *S*. *melongena* were high (Fig. 7d). In *L*. *esculentum*, maximum respiration rate was observed for T6 (20 µl g^−1^ dry wt) and minimum for T1 (17 µl g^−1^ dry wt) (Fig. 7c).

## Discussion

The outcomes of the current study reveal that vermicomposted fly ash on addition to soil enhanced the soil quality, improved the microbial and enzymatic activities and showed substantial increase in the growth and yield of tomato and brinjal. Perez-Murcia *et al*.^16^ and Iglesias and Jimenez^17^ stated that when composted materials are used as fertilizers, they should be completely stabilized to prevent negative growth effects caused due to oxygen depletion and nitrogen mineralization. The proportion of the compost added to soil is also important for preventing potential hazards. In the present investigation, the optimum concentration of VCF added to soil showing maximum growth was 15%. The experiments were also performed with 18% and 21% VCF however, above 15% of VCF, the plant growth and yield were observed to decline. The leaves synthesized more photosynthetic pigments and plants yielded more flowers and fruits. Leaves acquired a dark green colour because of increase in chlorophyll and carotenoid content. The plant pigments result in higher photosynthetic activity leading to enhanced growth and yield. Better growth and yield may also be owed to the improved nutrient content (N, P, K) in VCF. Mishra and Shukla^18^ reported about the existence of essential plant nutrients in fly ash.

The bulk density of the treatments was observed to be higher during sowing compared to harvesting. Pandey *et al*.^19^ and Goswami *et al*.^20^ observed a decline in bulk density and a rise in porosity and WHC on the application of FA to the soil. These results were consistent with the current study. Goswami *et al*.^20^ reported that vermicompost amendment improved soil structure by reduction of bulk density.

Porosity refers to the air space amongst the soil particles that is generally subjugated by water on availability^21^. Thus, increase in WHC on addition of VCF occurred because of greater space amongst the soil particles. EC shows positive correlation with pH thus representing the overall concentration of soluble anions and cations^22^. The EC values fall within the range desirable for the growth of crops^23^.

There were no significant differences between the metal concentration at the time of sowing and harvesting. The metal concentrations were within the permissible limits of soil for plant growth^24^ and were also lower than the critical limit of soil prescribed by^25^.

Dehydrogenase enzyme is extracellular in nature and mediates the oxidative phosphorylation process^26^ and is responsible for the microbial activity in biological environments. It is generally concerned with microbial energy metabolism in the gut of earthworms. High dehydrogenase activity in treatments comprising 15% VCF in both the plants might be due to huge quantities of readily degradable organic substrates in T6 accessible for the proliferation of microorganisms, that lead to augmented microbial activity and therefore to enhanced dehydrogenase activity. However, at later stages, the activity reduced because of substrate loss leading to diminished activity^27^. The variances in alkaline phosphatase activity of the treatments may be due to the changes in organo-phosphate complexes and variances in activity of microorganisms in each treatment. Phosphatase enzyme enhances agricultural attributes and can be changed to diverse forms of inorganic phosphorous (PNP), that can be assimilated by plants. Alkaline phosphate is linked along with the phosphorous (P) cycles and aids in the breakdown of organic phosphate esters^28^. High activity of alkaline phosphatase enzyme was maintained by the range of pH of VCF in which enzyme stayed active.

The bacterial populations were observed to be lower at the time of sowing compared to that of harvesting of *L*. *esculentum* and *S*. *melongena*. Maximum population of *Azotobacter*, Potash mobilizing bacteria and Phosphate solubilizing bacteria were observed for T6 in both the crops. *Azotobacter* shows a direct relation to the increase in the VCF, as it has higher population of bacteria^29^.

Siderophores are iron chelating compounds produced by bacteria and can act as biopesticide by preventing insect attacks on crops and plants^30^. HCN production by bacteria can be used as a pesticide for plants^31^. None of the bacterial strain in treatment comprising *L*. *esculentum* and *S*. *melongena* tested positive for HCN production. Plants cannot directly uptake the nitrate present in the substrate, hence the production of ammonia is an important PGP characteristic^32^.

Plants are unable to utilize phosphate present in the soil in its natural form. Phosphate solubilization makes the soil fertile and provides nutrients to the plants for agricultural effects^30^. Phosphorus is an essential micronutrient and is present in insoluble forms, thus converting them into soluble forms. This holds great significance for the plants.

MBC takes an efficient part in evaluating the microbial condition of the treatments and is perceptive to management systems and pollution^27^. Substrate health can be determined by MBC as it regulates nutrient cycling posing as a labile source of plant availability. The trend for MBC among the treatments was T6 > T5 > T4 > T3 > T2 > T1. This may be attributed to high organic matter and enhanced physico-chemical properties in treatments comprising VCF^33^. Moreover, microbial biomass carbon and respiratory activities are more in treatments comprising a higher concentration of VCF^27^.

Enhancement of seed germination with increased applications of VCF might be due to the good fertilizing ability of VCF applied at the rate of 15% to agricultural soil. Mishra *et al*.^34^ also reported that FA amendments caused significant improvement in the quality of the soil and germination percentage of crops.

The root length of *L*. *esculentum* was maximum for the treatment T6 during harvesting. Khan and Wajid^35^ reported that plant growth parameters such as root length and shoot length were found to increase with an increase in the concentration of FA to the soil. Significant (*p* < *0*.*05*) differences were obtained between the root length and shoot length of *S*. *melongena* in different treatments.

The number of leaves exhibited significant (*p* < *0*.*05*) differences between the various treatments at the different duration of sowing. Leaf production was high during the early stages of growth (30–60 DAS) but it decreased during later stages (30–90 DAS). This may be attributed to the fact that senescence occurs during later stages of growth^20^. Khan^36^ also observed that growing the tomato plants in the ash-soil mixture exhibited dense growth having more greener leaves.

An increase in fruit yield over control was observed throughout the treatments. The previous studies have also reported that augmentation of 40% FA to agricultural soil was useful for higher crop harvest, exceeding which had a hostile impact on crop yield^2,35^.

## Conclusion

This paper deals with the implications for the safe utilization of VCF in agricultural sectors. The rate of seed germination and plant growth were found to enhance with an increase in the application of VCF to the treatments in both *L*. *esculentum* and *S*. *melongena*. Fruit yield showed direct relation with VCF concentration and was maximum for the treatment comprising 15% VCF added to soil. The photosynthetic pigments (chlorophyll a and b, and carotenoids), levels of boron and total phenols were observed to reach a maximum in case of T6 while, they were minimum in case of T1. Thus, the VCF was observed to be a potent fertilizer when applied at the rate of 12–15% by weight to the agricultural soil leading to good crop growth and yield. Moreover, VCF is a biological fertilizer with reduced metal concentrations and enhanced N, P, K contents. It is thus necessary to utilize FA more effectively in the agricultural sector to reduce the burden of its disposal and exploit its physical and chemical properties completely, which are quite beneficial for soil and crop health.

## Materials and Methods

### Experimental setup and bio efficacy study

The VCF used in this study was obtained from a prior vermicomposting experiment carried out by Usmani *et al*.^10^, that involved mixing of coal FA (collected from Chandrapura Thermal Power Station) and cow-dung (collected from local area of Dhanbad) in the ratio of 1:3. The mixture was then subjected to vermicomposting using *Eudrilus eugeniae* species of earthworm for a duration of 90 days. The vermicompost (FA + CD; 1:3) attained by the above process was used in the current study as it was observed to be the best in terms of plant nutrient contents such as N, P and K and reduced metal concentrations compared to other tested mixtures. Seeds of *Lycopersicon esculentum* Mill. (Tomato) and *Solanum melongena* L. (Brinjal) were obtained from authorized vender of the local market. Soil sample was collected from the research field of IIT (ISM) Dhanbad, India.

The VCF used in this experiment was also compared with the prescribed guidelines of vermicompost provided by the Fertilizer Control Order, India (FCO) (Supplementary Table 1). The ecological risk assessment of metals in VCF was further determined by using the potential ecological risk index (PERI)^37^. The formulas used for the estimation of the coefficient of pollution (*Cf*^*i*^), potential ecological risk factor (*Er*^*i*^), and finally the risk index (PERI) are elaborated in Table 7. The trace element concentration in the VCF showed no obvious risks towards the environment based upon the *Cf*^*i*^, *Er*^*i*^ and PERI values (Table 8).

**Table 7:** Factors/Indices to determine potential ecological risk of vermicomposted fly ash to the environment.

Factor/Index	Formulas	Annotations	Threshold values
Coefficient of pollution (*Cf*^*i*^)	${{C}_{f}}^{i}=\frac{{{\rm{C}}}_{{\rm{M}}}}{{{\rm{C}}}_{{\rm{B}}}}$	*Cf*^i^: coefficient of pollution C_M_: concentration of individual metal in Vermicomposted fly ash (FA + CD; 1:3) C_B_: background concentration of the trace metal^62^ for Cr, Cu, Zn, Pb, As, Cd, Ni, and Co were 90, 45, 95, 20, 13, 0.3, 68, and 19 mg/kg	*Cf*^*i*^ = 0: none; *Cf*^*i*^ = 1: none to medium; *Cf*^*i*^ = 2: moderate; *Cf*^*i*^ = 3: moderate to strong; *Cf*^*i*^ = 4: strongly polluted; *Cf*^*i*^ = 5: strong to very strong; *Cf*^*i*^ = 6: very strong
Potential ecological risk factor (*Er*^*i*^)	$E{r}^{i}=C{f}^{i}\,x\,T{r}^{i}$	*Er*^*i*^: potential ecological risk factor of trace metal; *Tr*^*i*^: toxic metal response factor of trace metals. *Tr*^*i*^ for metals such as Zn, Cr, Cd, As, Cu, Pb and Ni are 1, 2, 30, 10, 5, 5, 5 as per^63^.	*Er*^*i*^ < 40: Low risk; 80 < *Er*^*i*^ < 160: considerable risk; 160 < *Er*^*i*^ < 320: high risk; *Er*^*i*^ > 320: very high risk^64^
Potential ecological risk index (PERI)	$PERI=\sum {{E}_{r}}^{i}$	$\sum {{{\rm{E}}}_{r}}^{i}$: Sum of potential ecological risk indices of all the heavy metals	PERI < 150: low risk; 150 < PERI < 300: moderate risk; 300 < PERI < 600: considerable risk; PERI > 600: very high risk^65^

**Table 8:** Potential ecological risk assessment of trace elements in vermicomposted fly ash to environment.

Metals	Coefficient of Pollution (*Cf*^*i*^)	Risk Evaluation as per *Cf*^*i*^	Potential ecological risk factor (*Er*^*i*^)	Risk Evaluation as per *Er*^*i*^	Potential ecological risk index (PERI) (Σ *Er*^*i*^)
Cu	0.05	No risk; *Cf*^*i*^ = 1	0.25	very low risk; *Er*^*i*^ < 40	Σ *Er*^*i*^ = Cu *Er*^*i*^ + Zn *Er*^*i*^ + Cd *Er*^*i*^ + Pb *Er*^*i*^ + Ni *Eri* + Cr *Eri* + As *Eri* Σ *Er*^*i*^ = 34.61 Indicating low risk towards environment
Zn	0.03	No risk	0.03	very low risk	
Cd	0.97	No risk	28.13	low risk	
Pb	0.60	No risk	3.00	very low risk	
Ni	0.18	No risk	0.90	very low risk	
Cr	0.05	No risk	0.10	very low risk	
As	0.22	No risk	2.2	very low risk	

The pilot experiment was performed in the research field of IIT (ISM) Dhanbad, Jharkhand. The VCF was mixed with agricultural soil at the rates of 3, 6, 9, 12 and 15% (w/w) (Supplementary Table 2). The treatment codes comprising different concentrations of VCF as amendments for pot experiments were as follows: T1 (Agricultural soil alone); T2 (Soil + 3% VCF); T3 (Soil + 6% VCF); T4 (Soil + 9% VCF); T5 (Soil + 12% VCF); T6 (Soil + 15% VCF). These soil samples were placed in earthen pots of 5 kg capacity (25 cm diameter). Control pot constituted only agricultural soil. To maintain drainage, a small perforation was made at the bottom of each pot. The study was carried out for a duration of 90 days (September 2016 to December 2016) and the plants were harvested after fruiting. During the growth period of crops, the temperature varied from 10–36 °C, humidity from 21–100% and air pressure showed variations from 996–1019 mbar. The detailed information about the growing environment of the crops over a duration of four months are presented in Supplementary Table 3. The experiment was performed in an entirely randomized block design with three replicates for every treatment.

### Estimation of physico-chemical characteristics of vermicomposted fly ash

The bulk density of VCF was evaluated by the soil core method^38^. Porosity was determined by dividing the volume of void spaces in the soil by the total volume of soil in the core and WHC by Keen-Raczkowski box method. pH (1:2.5 fly-ash: water) was determined using a digital pH meter (EI Model 101E). EC 1:2 (Fly-ash: water) was determined by digital conductivity meter (EI Model 612). Cation exchange capacity (CEC) was assessed through titration on switching the complex with ammonium ions and further titrating it using hydrochloric acid^39^. Total organic carbon was determined by the rapid dichromate oxidation method^40^. Total nitrogen by the CHNS elemental analyzer and total phosphorous by Phosphomolybdic blue colorimeter^41^. Exchangeable Ca and Mg by Ammonium acetate extractable method^42^ and estimated using Flame Atomic Absorption Spectrophotometer (FAAS, GBC AVANTA 3000). Metals like Cu, Zn, Cd, Ni, Fe and Cr were extracted by acid digestion and estimated using FAAS. Potassium, calcium, magnesium and manganese were determined by analytical methods suggested by^43^.

### Enzyme activity analysis

The assays of dehydrogenase and alkaline phosphatase enzymes were determined. Dehydrogenase activity was assessed by the procedure given by^44^. The dehydrogenase activity was measured by a UV-Spectrophotometer (UV-1800, Shimadzu, Japan) at wavelength of 485 nm. Alkaline phosphatase activity was measured by samples incubation with *p*-nitrophenyl phosphate at 37 °C for 1 h in an incubator^45^ and was measured at 480 nm in a spectrophotometer.

### Microbial biomass carbon determination

Soil microbial biomass carbon (MBC) was evaluated by sieving treatment sub-samples by the chloroform fumigation extraction (CFE) process as pronounced by^46^. The extracts obtained were examined for dissolved organic C by a Shimadzu TOC-L CSH with an OCT-L sampler (Shimadzu Corp., Kyoto, Japan) having 5X dilution as designated by^47^. Soil microbial biomass C was evaluated using the formula described by^48^.

1 $${\rm{MBC}}=({{\rm{C}}}_{{\rm{fumigated}}}\,{{\rm{C}}}_{{\rm{control}}})/\mathrm{kEC}$$

where, MBC: microbial biomass carbon; kEC: extraction coefficient

The extraction coefficients (kEC) used for carbon to determine MBC was 0.45 as per Potthoff *et al*.^49^ and Joergensen *et al*.^50^.

### Isolation of PGP bacterial strains

The different PGP bacterial strains were isolated in their respective selective medium by soil dilution pour plate technique at the time of sowing and harvesting of experimental crops. For PSB strain, the soil solution was grown in Pikovaskaya agar medium^51^, the colonies showing halo zone were initially considered as PSB strain. *Azotobacter* sp. was isolated on Ashby’s mannitol agar media (Himedia^®^, Mumbai, India), Potash mobilizing bacteria was isolated in Glucose yeast agar media (Himedia^®^, Mumbai, India) and the colonies showing potassium releasing zone were considered as potash mobilizing strain.

### Determination of PGP traits

Different PGP traits were determined for the treatments after harvesting of *L*. *esculentum* and *S*. *melongena* using standardized methods. IAA production was determined using the method employed by Gordon and Weber^52^. Siderophores production of the selected isolates were performed using Meyer and Abdallah^53^. Standard methods for hydrogen cyanide (HCN) and urea production were as per Lorck^54^ and Cappuccino and Sherman^55^, respectively. Phosphate solubilization was determined by Watanabe and Olisen^56^ method.

### Plant growth analysis

The bioefficacy study was grounded on germination of seeds, shoot and root length, dry and fresh weight of root and shoot, and the number of leaves at 30, 60 and 90 days after sowing (DAS). For treatment of seeds, collected seeds were superficially sterilized with 2% sodium hypochlorite for 3 mins and further washed 5 times with deionized water (1:1) under sterilized conditions^57^.

The rate of germination (R_G_) was calculated using the formula:

2 $${{\rm{R}}}_{{\rm{G}}}={\rm{\Sigma }}\,{{\rm{N}}}_{{\rm{i}}}/{{\rm{D}}}_{{\rm{i}}}$$

where N_i_ is the number of germinated seeds in each time and D_i_ is the time unit (day)^58^. To evaluate the growth parameters, plants were taken randomly and separated into the shoot and root. Shoot and root were rinsed to eliminate all soil particles and further dried in an oven at 70 °C for 3 days till constant weight was achieved^59^ for biomass analysis.

### Fruit and yield

Fruits were generally harvested weekly after attaining a mature stage. Picking was done 2–3 times as per the requirement. Fruit yield was assessed by counting and weighing all the fruits on individual plant.

### Photosynthetic pigments

The photosynthetic pigments like chlorophyll a, chlorophyll b and carotenoids were analysed from the leaves of *L*. *esculentum* and *S*. *melongena*. Fresh leaves weighing 0.5 g were homogenized in 20 mL of 80% acetone (Acetone: water v/v) in a pre-chilled mortar and pestle. The filtrate was centrifuged at 3000 rpm for 15 min in Janetzki refrigerated centrifuge Model K - 24 at 4 °C. The supernatant was decanted, and the volume was made up to 25 mL with 80% acetone. Care was taken to shield the chlorophyll extract from bright light. The optical density was measured at 480, 510, 645, and 663 nm wavelength using the spectrophotometer (UV-1800, Shimadzu, Japan). The amount of chlorophyll a, chlorophyll b and carotenoids were assessed using the formula described by^60^.

3 $${\rm{Chlorophyll}}\,{\rm{a}}=12.3{{\rm{D}}}_{663}-0.86{{\rm{D}}}_{645}\times {\rm{V}}\times {\rm{d}}\times 1000\times {\rm{W}}$$

4 $${\rm{Chlorophyll}}\,{\rm{b}}=19.3{{\rm{D}}}_{645}-3.6{{\rm{D}}}_{663}\times {\rm{V}}\times {\rm{d}}\times {\rm{1000}}\times {\rm{W}}$$

5 $${\rm{Carotenoid}}=7.6{{\rm{D}}}_{480}-1.49{{\rm{D}}}_{510}\times {\rm{V}}\times {\rm{d}}\times 1000\times {\rm{W}}$$

where, D = optical density at 480, 510, 645, 663 nm, respectively. V = volume of the chlorophyll extract in acetone (mL). d = light path length (cm). W = leaves fresh weight (g).

### Response parameters

The leaves were removed from the plants and leaf area was determined for all the leaves per plant. Fruit weights were measured and recorded. Fresh weights of plant shoot and root were documented. The plant parts were kept at 70 °C for 72 h and dry weights were also noted. Total Phenols in leaves were evaluated as per the methods explained by^61^. Data obtained were verified by statistical analysis.

### Photosynthesis and respiration rates

Photosynthesis and Respiration rate were determined for a distinct leaf bounded in a perspex chamber consisting of a leaf base fastened amid rubber gaskets to impart hermetic seals. The conditions were maintained as per the studies done by^18^. The removal rate of CO_2_ was assessed by a Grubb Parsons infrared gas analyzer and photosynthesis rate per unit leaf area was determined. Respiration rate was assessed using the volumetric method.

### Statistical analyses

The data on physico-chemical properties of the FA-soil mixtures were validated by Analysis of variance (One-way ANOVA followed by the *Tukey’s HSD* Test). The statistical strength of the data was determined by a volcano plot representing the expression of various FA amended soil parameters (MS Excel 16.0 v). Plant growth and yield were analyzed using Analysis of variance (One-way ANOVA) and least significant differences (L.S.D.) at *p~* < *0*.*05* were estimated. The mean values of these parameters were compared by means of Duncan’s multiple range test (DMRT) at *p* ≤ 0.05 level of significance for the column factor. Pre-and post-plantation soil study was determined by paired-sample *t* test using SPSS software package 20.0 version. Raw data on yield of plant was assessed by curvilinear regression to examine responses relating to the FA concentration.

## Supplementary information

Supplementary Material
