# Supplementary material for: Enhanced soil fertility, plant growth promotion and microbial enzymatic activities of vermicomposted fly ash
Source: Sci Rep. 2019 Jul 18;9:10455. doi: 10.1038/s41598-019-46821-5 (PMC6639538; doi:10.1038/s41598-019-46821-5)
Supplement: Supplementary file 1 — Supplementary Material [file 41598_2019_46821_MOESM1_ESM.doc]

***Enhanced soil fertility, plant growth promotion and microbial enzymatic activities of vermicomposted fly ash***

**Zeba Usmani**^a^**, Vipin Kumar**^a^***, Pratishtha Gupta^a^, Gauri Gupta**^a^**, Rupa Rani^a^, Avantika Chandra**^a^

*^a^* *Laboratory of Applied Microbiology*

*Department of Environmental Science and Engineering*

*Center of Mining Environment*

*Indian Institute of Technology (Indian School of Mines) Dhanbad*

*Dhanbad, Jharkhand, India*

**Corresponding Author; Email:* [*vipinmicro1@*](mailto:vipinmicro1@)*iitism.ac.in; Phone: +91 9471191352*

**Supplementary Information:**

**Supplementary Table 1.** Comparison of vermicomposted FA with prescribed vermicompost limits given by Fertilizer Control Order (FCO), India.

| **Parameters** | **Vermicomposted Fly ash** | **Vermicompost by FCO** |
| --- | --- | --- |
| Moisture percent | 18.76 | 15-25 |
| Colour | Dark brown | Dark brown to black |
| Odour | No foul odour observed | Absence of foul odour |
| Particle size | 94% of material passed through 4.0 mm IS sieve | Minimum 90% Material should pass through 4.0 mm IS sieve |
| Bulk Density (g/cm3) | 0.87 | 0.7-0.9 |
| Total Organic Carbon (%) by weight | 8.76 | 18.00 |
| Total Phosphate (P_2_O_5_) | 1.25 | 1.0 |
| Total Potassium (K_2_O) | 3.5 | 1.0 |
| As (mg/kg) | 2.87 | 10.00 |
| Cd (mg/kg) | 0.29 | 5.00 |
| Cr (mg/kg) | 4.26 | 50.00 |
| Hg (mg/kg) | bdl | 0.15 |
| Ni (mg/kg) | 12.25 | 50.00 |
| Pb (mg/kg) | 12.08 | 100.00 |
| Cu (mg/kg) | 2.25 | - |
| Zn (mg/kg) | 3.18 | - |

IS: Indian Standards; bdl: below detection limit; (-): not provided

Vermicomposted fly ash data obtained from the study conducted by Usmani et al. ^(10)^.

**Supplementary Table 2.** Treatments comprising different concentration of vermicomposted fly ash as amendments to soil for pot experiments.

| **Treatments/ Codes** | **Description**  (% by weight) |
| --- | --- |
| T1 | Agricultural soil alone (control) |
| T2 | Soil + 4 % Vermicomposted fly ash (VFA) |
| T3 | Soil + 8 % VFA |
| T4 | Soil + 12 % VFA |
| T5 | Soil + 16 % VFA |
| T6 | Soil + 20 % VFA |

VFA: Vermicomposted fly ash; Weight of soil taken: 5 kg

**Supplementary Table 3.** Growing environment of crops from September 2016 to December 2016

| *September (2016) Growing Conditions for crops* | | | |
| --- | --- | --- | --- |
|  | **Temperature (°C)** | **Humidity (%)** | **Pressure (mbar)** |
| **Lowest** | 22 | 43 | 997 |
| **Highest** | 36 | 98 | 1010 |
| **Average** | 28 | 85 | 1003 |
| *October (2016) Growing Conditions* | | | |
| **Lowest** | 19 | 30 | 1001 |
| **Highest** | 34 | 97 | 1016 |
| **Average** | 27 | 73 | 1007 |
| *November (2016) Growing Conditions* | | | |
| **Lowest** | 14 | 23 | 1006 |
| **Highest** | 35 | 92 | 1018 |
| **Average** | 23 | 67 | 1013 |
| *December (2016) Growing Conditions* | | | |
| **Lowest** | 10 | 21 | 1009 |
| **Highest** | 30 | 100 | 1019 |
| **Average** | 19 | 67 | 1014 |
